# Innovative Approaches to Active and Healthy Ageing: Campania Experience to Improve the Adoption of Innovative Good Practices

**Published:** 2019-01-06

**Authors:** V De Luca, G Tramontano, C Del Giudice, I Grimaldi, R Romano, I Liguori, M Carpinelli Mazzi, N Di Carluccio, PA Riccio, P Speranza, A Iavarone, P Abete, A Postiglione, M Cataldi, C Vallone, F Giallauria, A Cittadini, M Triggiani, S Savastano, E Menditto, L Leonardini, A Colao, M Triassi, G Iaccarino, A Postiglione, E Coscioni, M Illario

**Affiliations:** 1 Unità Operativa Semplice Ricerca e Sviluppo, Azienda Ospedaliera Universitaria Federico II, Naples, Italy; 2 Dipartimento di Scienze Mediche Traslazionali, Università degli Studi di Napoli Federico II, Naples, Italy; 3 Dipartimento di Medicina Clinica e Chirurgia, Università degli Studi di Napoli Federico II, Naples, Italy; 4 Consorzio Farmacie Servizi (COFASER), Sarno SA, Italy; 5 Associazione Progetto Alfa Onlus, Marigliano NA, Italy; 6 Unità Operativa Complessa Gestione Affari Generali, Azienda Ospedaliera Universitaria Federico II, Naples, Italy; 7 Unità Operativa Complessa di Neurologia, Azienda Ospedaliera Specialistica Dei Colli - Monaldi-Cotugno-CTO, Naples, Italy; 8 Dipartimento di Neuroscienze, Scienze Riproduttive ed Odontostomatologiche, Università degli Studi di Napoli Federico II, Naples, Italy; 9 Dipartimento di Sanità Pubblica, Università degli Studi di Napoli Federico II, Naples, Italy; 10 Dipartimento di Medicina, Chirurgia ed Odontoiatria, Università degli Studi di Salerno, Salerno, Italy; 11 Dipartimento di Farmacia, Università degli Studi di Napoli Federico II, Naples, Italy; 12 Progetto Mattone Internazionale Salute, Ministero della Salute, Rome, Italy; 13 Dipartimento di Scienze biomediche avanzate, Università degli Studi di Napoli Federico II, Naples, Italy; 14 Direzione Generale per la Tutela della Salute e il Coordinamento del Sistema Sanitario Regionale (DG04), Regione Campania, Naples, Italy; 15 Struttura Dipartimentale di Chirurgia dell’Aorta Ascendente e Toracica, Azienda Ospedaliera Universitaria Ospedali Riuniti San Giovanni di Dio e Ruggi d’Aragona, Salerno, Italy; 16 Unità Operativa Dipartimentale Promozione e potenziamento di programmi Health Innovation (DG04), Regione Campania, Naples, Italy

**Keywords:** Innovation, Ageing, Information and Communication Technology, Public Health

## Abstract

The demographic projections on the European population predict that people aged over 60 will increase by about two million/year in the next decades. Since 2012, the Campania Reference Site of the European Innovation Partnership on Active and Healthy Ageing supports the innovation of the Regional Health System, to face up demographic changes and sustainability. Campania Reference Site provides the opportunity to connect loco-regional stakeholders in social and health care services (universities, healthcare providers, social services, local communities and municipalities), with international organizations, in order to adopt and scale up innovative solutions and approaches. This paper describes the building process of Campania Reference Site and the main results achieved, that have been allowing it to become a hub for open innovation in the field of active and healthy aging at regional, national and international level.

## I. INTRODUCTION

The European population aged over 60 will increase by about two million/year in the next decades, impacting on public budgets [1]. Campania faces the burden of an aging population, with a cluster over 65 of 1.075.405 at the same extent of the other EU countries (ISTAT 2018). The current economic crisis has caused a reduction in the services provided to European citizens, creating shortages in health care and social services. In Campania, the effects of the crisis should be added to a pre-existing low level of socio-economic development. Campania presents a very modest quality of services, ranking at the 20th place among the Italian regions, with particular reference to services for frail individuals and older adults. The current Regional Health Plan (*Piano Sanitario Regionale 2011–2013, Decreto Commissario Ad Acta n.22/2011*) highlights the need to balance the distribution of services between the hospital and territorial levels, moving resources (economic, human and technological) to the territory, to manage increasing chronic diseases, comorbidities and population ageing.

The most important process that has been influencing Campania system’s capacity to improve health outcomes has been the Contingency plan, that was established in March 2007 (Regional Resolution n.460/2007) and is still in place. Under a contingency plan only ordinary expenses are allowed, and the turnover of the human resources is blocked, with a subsequent progressive reduction and ageing of the workforce for the physiological retirements, that is not replaced.

The impact of the recovery plans has been the achievement of budget balance, that started from a unbalance of 750ME/year. The contingency plans implemented a forced, top-down re-organization of the Regional Health System, reducing the number of local health agencies from 15 to 7, concentrating purchasing activities in regional or supra-organizational entities and integrating some independent hospitals to local health agencies.

On the verge of Campania exit from the contingency plan, it is pivotal that novel tools and processes are implemented to strengthen the system’s capacity to improve health outcomes, avoiding the paradox effect of horizontal cuts, that mostly affect prevention and health promotion activities, and the risk of a novel budget unbalance due to the ageing population.

## II. THE INNOVATION FRAMEWORK FOR HEALTH IN THE EU: THE EIP ON AHA

The opportunity to enhance change management in health service provision can take advantage of the European Innovation Partnership on Active and Healthy Aging (EIP on AHA) partner’s good practices and experience in improving system sustainability. The EIP on AHA is an initiative launched by the European Commission (EC) to foster innovation and digital transformation of health and care in the field of active and healthy ageing [2,3]. The EIP on AHA brings together all the relevant actors at EU, national and regional levels across different policy areas to handle the societal challenge of the active and healthy ageing. The Strategic Implementation Plan (SIP) of the EIP on AHA set the goal of increasing the healthy lifespan of EU citizens by 2 years by 2020. The SIP establishes the vision, the strategy and a concrete operation plan, within 3 main vertical pillars: prevention and early diagnosis; care and cure; and active ageing and independent living [4].

The modalities to cooperate to the implementation strategies of the EIP on AHA are: 1) to become a Reference Site; 2) to take part to the Action Groups.

The acknowledgement of the Reference Site (RS) of the EIP on AHA was awarded by the European Commission in 2012, after a “peer review” evaluation, to the Research and Development (R&D) Unit of *Azienda Ospedaliera Universitaria* (AOU) Federico II, that applied, on behalf of the Campania Region, to an international call issued by the EC. Subsequently, the RS also obtained recognition from the Campania Region (Regional Resolution n.622/2012), that established a specific Coordination Team to pursuit the objectives of the SIP of the EIP on AHA. In 2016, the R&D Unit of the AOU Federico II has re-submitted the application to RS of the EIP on AHA call, achieving the third star at the RS Awards, based on the results achieved during its coordination. The recent Regional Resolution n.221/2017 attributed the coordination of the Campania RS to the Health Innovation Division of the General Directorate for Health Protection and the Coordination of the Regional Health System of Campania Region (DG04), and established an interdisciplinary working group to ensure the harmonization of regional planning in the health sector, identifying the AOU Federico II as a member.

The vision of Campania RS is stimulating and supporting the set-up of a local ecosystem to face the challenge of an ageing population with a life-course approach, where innovations are exploited to improve health outcomes, quality of life and sustainability of social and health services.

Campania RS supports the adoption of iterative models of implementation of the innovations in all domains of AHA, to ensure adoption, facilitate the transfer of the innovation to the market, and integrate the innovative solutions with existing ICT platforms, ensuring interoperability.

RS’s ambition is connecting innovations with end-users by increasing health equity, health promotion and disease prevention.

Since 2012, Campania RS collaborated with European and regional stakeholders for the adoption of a “Quadruple Helix” model [5], in a collaborative effort to meet the challenges of Campania in the field of AHA.

The RS involved many actors and collaborative efforts have been activated to identify, test, implement and scale innovative solutions, such as:

- Local Health Authorities, Hospitals;
- Campania Regional Health Authority;
- Higher School Institutes, Universities, Research Centers;
- Municipalities;
- Non-profit organizations, Industry.

RS cooperate with other European and national networks, as the EIP on AHA Reference Site Collaborative Network (RSCN), European Health Telematics Association (EHTEL) and Programma Mattone Internazionale Salute (ProMIS), expanding the possibilities for regional stakeholders to participate in international projects and coordinating the activities of the various thematic working groups [6].

The EIP on AHA is structured into 6 Action Groups (AG):

- A1) Prescription and adherence;
- A2) Personalized health management and falls prevention initiatives;
- A3) Lifespan Health Promotion and prevention of age-related Frailty and Disease;
- B3) Integrated care for chronic diseases;
- C2) Development of interoperable “independent living” solutions to prevent old people isolation;
- D4) Promotion of age-friendly environments;

The Action Groups focus on sharing information and solutions on how to undertake joint actions to achieve the common goal of improving the quality of life of the aging population.

Campania RS contribution to A1 AG objectives has been carried out by a joint effort of AOU Federico II, University of Salerno and *Centro Interdipartimentale di Ricerca in Farmacoeconomia e Farmacoutilizzazione* [Interdepartmental Research Centre for Pharmacoeconomics and Drug use] (CIRFF) to the “*Farmaci Rivisti Insieme: Empowerment Nelle Diverse Discipline*” [Joint review of drugs: empowerment in different disciplines] (FRIENDD) multidisciplinary focus group, including clinical specialists, pharmacologists, pharmacists and ICT experts, with the mission to implement innovative approaches to optimize drug therapy in multimorbid older patients. To address these points a retrospective observational pilot study has been implemented to establish to which extent the drug therapy of individual patients is modified by the hospital medical team. To address this point, the drug therapies at the time of hospital admission and discharge have been compared in the medical records of the patients admitted to the Geriatrics and Cardiology Wards of the AOU Federico II during the last five years. Specifically, type and number of the prescribed drugs and number and severity of potential drug interactions have been evaluated. In order to get information about possible age-related differences, the medical records of all patients older than 18 have been evaluated. Potential drug interactions have been evaluated with the Medscape Interaction Checker online resource (http://reference.medscape.com/drug-interactionchecker).

708 medical records have been examined. 299 patients were females. 359 patients were older than 65 (157 females and 201 males). In the whole population, the average number (95% CI) of drugs at the time of hospital admission and of discharge was 5.0 (0.254) and 6 (0.265) respectively, and this difference was statistically significant. When only older patients were considered, 6 (0.348) and 7(0.365) drugs were taken at the admission and discharge, respectively. In older adults we observed a higher number of potential drug interactions both at admission - 4(0.767) and at discharge - 5(0.876) - but these values were not significantly different from those observed in patients younger than 65 [7, 8].

The A3 AG aims to prevent functional decline and frailty by accelerating innovation in the prevention and management of frailty by all relevant stakeholders. A3 partners focus on synergic approaches that can be integrated to prevent frailty and disability along the domains of:

- Food and Nutrition
- Physical Activity
- Frailty
- Functional decline
- Cognitive decline
- Caregivers.

Since 2013, Campania has been actively participating in the coordination team of the AG A3, contributing to the development of reference documents of the group [9–13].

The activity of Campania RS in the A3 AG integrates health promotion, disease prevention, screening, early detection and monitoring of frailty through the assessment of older adults along different dimensions. Campania RS effort is focused on the set up of new service models for older people, where screenings for frailty are linked to targeted novel interventions to prevent it, as well as to prevent frailty along the entire lifecourse. As coordinator of the “Food and Nutrition” Action Area, Campania RS contributed to outline the A3 AG strategy for the primary nutritional approach, which outlined a step-wise approach to malnutrition. This approach links assessment to adequate interventions (primary/secondary/tertiary), and is aimed to implement innovative tools for effective prevention, detection, and treatment measures [14–18].

## III. THE INVOLVEMENT OF CAMPANIA STAKEHOLDERS IN DESIGN, PILOTING AND SCALING-UP GOOD PRACTICES

In 2016 the Directorate-General for Communications Networks, Content and Technology (DG CNECT) of the European Commission initiated the Transfer of Innovation Twinning Support Scheme through a call to contribute to the European scaling-up strategy of the EIP on AHA [19, 20].

Allergic Rhinitis and its Impact on Asthma (ARIA) develops a multisectoral pathway for rhinitis and related multimorbidity in older adults. The project is developed through a smartphone app that allows assessment of rhinitis, and uses clinical decision support system (CDSS) [21,23]. The goal of the scale-up in Campania is to accelerate the adoption of the tool by users through the network of local health agencies and pharmacies. The ARIA initiative aims to develop an inter-professional care models (Pharmacists, GPs, Specialists) for an integrated management of patients affected by allergic rhinitis.

The Quick mild cognitive impairment screen (QMCI) is a rapid and reliable tool that has shown good psychometric properties to distinguish normal people from subjects with MCI [24–28]. Campania RS multicenter study has been aimed to validate the Italian version of the QMCI (QMCI-I) and to obtain normative data.

Further study in Italian patients with MCI will better clarify the diagnostic properties of the QMCI-I. To address the question if the multi-domain structure of the tool could be useful to classify the several subtypes of MCI [29].

Campania RS developed a pilot study for the adoption of the tool of tele-rehabilitation, Telerevalidatie.nl®, to evaluate the adoptability of the tool in patients with Chronic Heart Failure and Cystic Fibrosis. Telerevalidatie.nl® is an ICT Platform that supports rehabilitation at home, allowing the patient to receive a personalized training program with tutorial videos and to track their training progress and physical activity during all day. The platform aims to improve adherence to cardiac and respiratory rehabilitation [30–33].

According to the vision of the EIP-AHA A3 AG Food & Nutrition [18] an ICT supported personalized interventions has been implemented to take advantage of validated screening, assessment and monitoring tools, aimed at improving food intake in older adults. A digital Modular Gastrological Platform (MGP) has been developed to facilitate inter-professional efforts. MGP focuses on supporting workflows in the Primary and Secondary Care Level, grounded in capacity/knowledge building of the chef workforce.

“*Assistenza Domiciliare per Dimissioni Protette*” [Home care for Protected discharge] (ADD Protection) model of the University of Salerno in collaboration with the Ruggi University Hospital, is an innovative practice consisting in an ICT-based home monitoring system provided as a service by a private company of home care, that allows the hospital staff to follow the patients at home, as if the patient was still in the hospital. The data collected at the patient’s home are made available to the staff of the hospital through a web-based platform, which feeds the hospital Electronic Health Record (EHR) of the patient.

## IV. RESEARCH AND INNOVATION COLLABORATIVE PROJECTS

Personalised ICT Supported Service for Independent Living and Active Ageing (PERSSILAA) is a European project financed by Seventh Framework Program (FP7) aimed to assess and monitoring of frailty in community dwelling older adults. The new services are offered to older adults through local community service, and are seamlessly integrated with health care services. This new multimodal service model, focusing on nutrition, physical, cognitive and social domains, is supported by an interoperable ICT service infrastructure and gamification. PERSSILAA develops service modules for screening, monitoring and training. Screening focuses on the use of accessible and user-friendly screening instruments to get an overall picture of an individual’s nutritional, functional and cognitive state. Monitoring involves unobtrusive ambulant monitoring of every day functioning and its change in time in terms of being physically active, performance of cognitive demanding tasks, nutrition behavior and demand of care. PERSSILAA focuses on creating awareness, motivating older adults to and supporting them in practicing and maintaining healthy behaviors as much as possible by themselves via community services but when needed proactively supported by cares and professionals. Preliminary results obtained by PERSSILAA assessment provide a sample of the distribution of frail older adults in Campania for the different domains that have been assessed [34–36].

SUNFRAIL (Reference Sites Network for Prevention and Care of Frailty and Chronic Conditions in community dwelling persons of EU Countries) is a European project aimed at the design and validation of an innovative and integrated model for the identification, prevention and management of frailty and multi-morbidity. The SUNFRAIL Model of Care provided the early identification of frailty and its risk factors in order to prevent its worsening and adverse outcomes. For this purpose, SUNFRAIL tool has been designed including 9 questions selected from evidence-based tools already adopted in health services to identity frailty according to the bio-(physical), psycho- (cognitive and psychological) and social domains.

Campania Region, *via* AOU Federico Il, administered the SUNFRAIL tool in its “Geriatric Evaluation Unit” on a cohort of 111 non-institutionalized patients who underwent a Geriatric Comprehensive Assessment with several multidimensional tools including the evaluation of cognitive impairment (Mini Mental State Examination) depression (Geriatric Depression Scale) disability (Basic and Instrumental Activity Daily Living), and comorbidity (Cumulative Illness Rating Scale). Successively, Italian Frailty Index (IFi) [37] and SUNFRAIL tool were administered. A linear regression analysis between these two tools was performed and a good linear correlation (r=0.67, p<0.001) was found. The good linear relationship between the two tools demonstrated that SUNFRAIL tool can be used for frailty evaluation, probably obtaining the same results as the IFi, and therefore administered for primary prevention in a fast way and by non-geriatricians or specialists.

“Beyond Silos” is a project of the University of Salerno aimed to address the issue of de-hospitalization by designing and implementing a network of services and facilities that is tailored upon the local context, to support the reduction of the cost of avoidable hospitalization by implementing an efficient and effective ICT system. These devices allowed the detection, transfer and monitoring of the clinical parameters that were stored at the hospital servers, supporting the hospital doctors to take medical decisions, in order to adjust the care pathway. Such a system integrates the function of hospitals for chronic diseases through a health management model network that identifies a range of facilities, professionals, equipment and tools [38–40].

“A comprehensive approach to promote a disability-free Advanced age in Europe: the ADVANTAGE initiative” (ADVANTAGE) is a Joint Action with 22 Member States (MS) and 35 organizations involved. Partners work together to summarize the current State of the Art of the different components of frailty and its management. The final output will be the “Frailty prevention approach” (FPA), a common European model to tackle frailty and indicate what should be prioritized in the next years at European, National and Regional level and on which to base a common management approach of older people who are frail or at risk of developing frailty in the EU. The identification of the core components of frailty and its management should promote the needed changes in the organization and the implementation of the Health and Social Systems.

“Stimulating Innovation Management of Polypharmacy and Adherence in The Elderly” (SIMPATHY) is an EU financed project aimed at stimulating innovation across the EU in the management of appropriate polypharmacy and adherence in older adults. Through a programme of work and stakeholders engagement, case studies have been performed in a range of different healthcare environments, including EIP on AHA RSs, providing a framework and political-economic basis for an EU wide benchmarking survey of strategies being employed for polypharmacy and non-adherence management. [41–45].

## V. INNOVATIVE PROCUREMENTS

Horizon 2020, the EU framework program for research and innovation, introduced two innovative forms of public procurement: Public Procurements for Innovative Solutions (PPI) and Pre-Commercial Procurements (PCP). Public Procurement for Innovative Solutions (PPI) means a procurement procedure in which contracting authorities act as customers for launching innovative goods and services that are not yet available on a large-scale commercial basis. Pre-commercial procurement (PCP) is a public procurement of innovation, developed specifically for the purchase of Research and Development services rather than for real goods and services. PPI and PCP cover a wide range of the industrial market through all phases of development - from research to the final product - giving to public buyers the opportunity to influence the market towards innovative solutions. Campania RS is very active in the field of Innovative Procurements, as several projects have received funding from European and national resources.

“Procuring innovative ICT for patient empowerment and self-management of type 2 diabetes mellitus” (PROEMPOWER) is a PCP project aimed to purchase Research and Development services in order to develop a novel ICT tool able to early diagnose diabetes, to facilitate the lives of people with type 2 diabetes, supporting them in their daily lives and allowing the health organizations to manage clinical data to prevent and intervene in case of acute events.

“*Ricerca e sviluppo di un sistema integrato per la gestione della multimorbidità dell’anziano attraverso l’aderenza alle prescrizioni*” [Research and development of an integrated system for managing the multimorbidity in older adults through prescription adherence] (ADCARE) is a PCP project aimed to the research and development of an integrated model, supported by ICT, to increase the compliance to therapy in chronic disease older adults patients from the hospital access to the follow-up. In particular, the project’s IT system will harmonize the procedures for prescribing and monitoring of therapies from the hospital to the patients’ home.

“*Modelli e strumenti per la gestione efficace delle nuove tecnologie biomedicali in organizzazioni sanitarie complesse*” [Models and tools for the effective management of new biomedical technologies in complex healthcare organizations] is a PCP project aimed to design and validate a solution for an effective management of the life cycle of new biomedical technologies in complex health organizations. The project will provide innovative solutions to support the investments planning, technology detection, management and disposal of biomedical technologies.

## VI. CONCLUSIONS

Campania RS is an example of structured networking, with a flexible approach that aims to bring Campania closer to Open Innovation [46].

Innovation needs ideas, products, talents that may not always be available within the region. What happens outside should be considered valuable when consistent with the organization of the regional health system needs. Adopting the Open Innovation paradigm mitigates the main disadvantages of producing innovation: high cost, need for vertical skills, longer time to market. Being a RS offers four key benefits to Campania region:

1. Stimulates innovation on AHA, a key theme for the sustainability of Campania health system, with external inputs: products, services and innovative approaches.
2. It gives access to validated technologies on which to invest.
3. It increases the skills and capacity of management and of the regional health system internal resources, in an increasingly “digital” market scenario.
4. Fosters and strengthens inter-organizational and multidisciplinary collaborations at the regional, national and international levels.

The collaboration between AOU Federico II and Campania Region in the management of the RS has been pivotal to ensure the extension of the benefits to all health organizations in the region. The connection to the ProMIS network stimulated the set up of the ProMIS regional working group, where the Health Innovation Division has been grounding the structured exchange and exploitation of the validated experiences and good practices, stimulating the scale up and the internationalization of all the actors of the system.

## Figures and Tables

**Figure 1:**
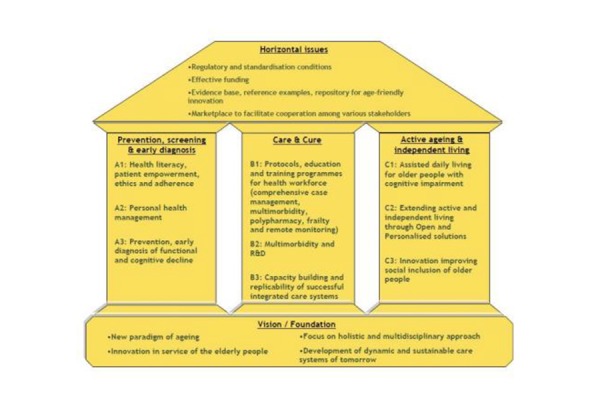
The priority actions of the Strategic Implementation Plan

**Figure 2:**
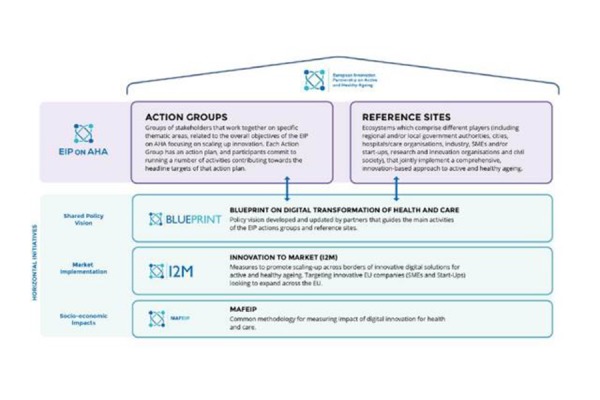
The structure of the European Innovation Partnership on Active and Healthy Ageing
